# Integrating Forensic Autopsies with Proteomic Profiling for Suicide Risk Assessment: A Comprehensive Review of Literature

**DOI:** 10.2174/011570159X344453241129073214

**Published:** 2025-01-20

**Authors:** Ibrahim Hasan Al-Habash, Asma Mahmoud Alshaeb, Viktorija Belakaposka Srpanova, Djordje Alempijevic, Milica Keckarevic-Markovic, Monica Concato, Davide Radaelli, Stefano D’Errico

**Affiliations:** 1 Forensic Medicine Department, Mutah University, Karak, Jordan;; 2 Forensic Medicine Center, Ministry of Interior, Doha, State of Qatar;; 3 Institute for Forensic Medicine, Criminalistic and Medical Deontology, Medical Faculty, Skopje, R. Macedonia;; 4 Institute of Forensic Medicine 'Milovan Milovanovic', Faculty of Medicine, University of Belgrade, Belgrade, Serbia;; 5 Department of Biochemistry and Molecular Biology, Faculty of Biology, Center for Applied and Forensic Molecular Genetics, University of Belgrade, Belgrade, Serbia;; 6 Department of Medical Surgical and Health Sciences, University of Trieste, Trieste, Italy

**Keywords:** Proteomics, biomarkers, suicide, postmortem, autopsy, risk assessment, profiling

## Abstract

**Background:**

Suicide is a major global public health concern that affects people of all ages, with over 700000 individuals intentionally ending their lives every year. Suicide is a multi-factorial event related to multiple risk factors interlocking with each other, among which neurobiological factors are considered to be an objective measure of the incidence of this phenomenon and can be used as a measurable tool for evaluating suicidal tendencies.

**Objective:**

The aim of this study is to thoroughly examine available data and assess candidate proteins as prospective biomarkers for predicting suicides and ascertaining the manner of death in forensic cases.

**Methods:**

An electronic search was conducted on PubMed, Science Direct Scopus, and the Excerpta Medica Database. The systematic review adhered to PRISMA guidelines and encompassed case series, prospective and retrospective studies, and short communications published in English. The focus was on proteomics and suicide, specifically, those studies where researchers conducted human proteomic analyses on specimens obtained from individuals who completed or attempted suicide.

**Results:**

A total of 14 studies met the inclusion criteria, resulting in a dataset of numerous candidate protein biomarkers. These include tenascin-C, potassium voltage-gated channel subfamily Q member 3, vimentin-immunoreactive astrocytes, glutathione S-transferase theta 1, iron transport proteins, A-crystallin chain B, manganese superoxide dismutase, glial fibrillary acidic protein, various glycolytic pathway proteins, 14-3-3 eta and 14-3-3 theta proteins, specific cytoskeleton proteins, C-reactive protein, serum amyloid A protein 1, extrinsic coagulation pathway proteins, the vacuolar-type proton pump ATPase subunit, plasma apolipoprotein A-IV, and ER stress proteins. These proteins are proposed as a panel of biomarkers to be evaluated in conjunction with other clinical predictors of suicide.

**Conclusion:**

This review aims to provide a comprehensive summary of all proteomic studies conducted on cases of attempted or completed suicide. By doing so, it seeks to bridge existing gaps in knowledge and pave the way for future investigations. The ultimate goal is to potentially identify a suicide biomarker.

## 1 INTRODUCTION

Suicide is a major global public health concern that affects people of all ages [1]. According to the World Health Organization (WHO), over 700000 individuals intentionally end their lives every year [2]. A significant problem related to this issue is the consistent increase in the suicide rate globally [3].

Suicide is a multi-factorial event related to multiple risk factors interlocking with each other [4]. These risks include psychological, clinical, biological, social, and environmental factors [5, 6]. The identification of these factors in individuals prone to suicide reveals, however, their subjective nature. Among them, the neurobiological factor is considered to be an objective measure of the incidence of this phenomenon and can be used as a measurable tool for evaluating individuals, especially psychiatric patients with suicidal tendencies [7]. Multiple neurobiological studies have been conducted to assess the susceptibility to suicide in individuals [8]. These studies include genomics, transcriptomics, epigenomics, proteomics, metabolomics, and microbiomics [9]. Even though these studies have been used widely in the medical fields, their use in the assessment of mental disorders and suicide is still limited [10, 11].

The term proteomics refers to the systematic analysis of proteins for their identity, quantity, and function [12]. This evaluation encompasses the complete set of proteins expressed by a cell or tissue under specific conditions, including those resulting from alternative gene splicing and posttranslational modifications [8]. It is crucial to assess the role of proteins in any entity since genetic information about proteins often provides incomplete information about their expression in cells or tissues due to differential gene expression and frequent regulation at posttranscriptional and posttranslational levels [13]. In addition, proteomes are thought to be dynamic and susceptible to both intra- and extracellular influences. Factors that are related to suicidal behavior may affect the proteome, causing alterations in protein expression. Consequently, the appearance of specific proteins or protein modifications can serve as a tool for the evaluation and possible prediction of suicidal behavior [5].

Some studies revealed an abnormal proteome in the pathway of neurotransmission and synaptic signaling, such as the endocannabinoid signaling pathway, γ-aminobutyric acid (GABA) receptor signaling pathway [14], KCNQ-type potassium channels (potassium voltage-gated channel subfamily Q member), response element binding protein (CREB) signaling, noradrenergic and dopaminergic signaling [15], and serotonergic and melatonin signaling associated with suicide [8]. Proteins involved in neuroglial function, neurodegeneration, and oxidative stress neuronal injury are also found to be altered in cases of suicide and suggested to be related to the phenomenon [5]. Proteome analysis of suicidal individuals also indicated changes in other proteins linked to biological structures and functions, including metabolism, the cytoskeleton, the redox system, synaptic function, and proteolysis [16].

Proteomics and psychiatric disorders have been extensively studied; however, there is a notable lack of reviews focusing specifically on proteins associated with suicide. In this study, suicide-related proteomics is reviewed. Proteins whose alterations are found to be associated with suicide can be considered as possible suicide biomarkers, indicating the probability or severity of suicidal tendencies or assisting in determining the manner of death in cases lacking suggestive signs of intent. This information may be useful in the fields of forensics, clinical psychiatry, and pharmacology.

## 2 MATERIALS AND METHODS

### 2.1 Eligibility Criteria

The present systematic review included all case series, retrospective and prospective studies, and short communications published in English that focused on proteomics and suicide, in which the researchers conducted a proteomic analysis for specimens obtained from suicidal completers or attempters. The search was limited to human studies only after revising the studies conducted on animals for evaluation.

The criteria for exclusion from this research were as follows. Articles not published in English were excluded, as were studies involving animal models rather than human subjects. Additionally, proteomic analyses not specifically related to suicidal behavior or attempts were omitted. Studies that failed to specify the exact proteins analyzed were also excluded. Systematic reviews were not considered, and sources categorized as unidentified or grey literature were excluded as well.

### 2.2 Search Criteria and Critical Appraisal

A systematic literature search was undertaken, followed by a critical evaluation of the gathered studies. By utilizing PubMed, Science Direct Scopus, and the Excerpta Medica Database (EMBASE), all available data was covered from the inception of these databases up to May 1, 2024. The search terms employed were “proteomics” AND “suicide”, restricted to titles, abstracts, and keywords. Studies involving proteomic analysis unrelated to suicidal cases or attempts were excluded from consideration.

Each selected paper underwent thorough bibliographic review and cross-referencing to find other pertinent research. Based on the Reporting Items for Systematic Reviews and Meta-Analyses (PRISMA) 2020 Guidelines [17], a methodological assessment of every study was carried out. Data extraction and study selection were required for data collection. A.S. and I.H., two researchers, separately looked over the articles whose titles or abstracts seemed pertinent, and they chose the ones that included a proteome analysis for suicidal cases and attempters. The two researchers used a consensus approach to settle disagreements on eligibility. Unpublished, unidentified, or grey literature was not included. Two researchers (A.S. and M.C.) carried out the data extraction, while two other researchers (D.R. and I.H.) confirmed the results. Since this study did not involve human subjects, institutional review board permission was not required.

## 3 RESULTS

### 3.1 Search Results and Included Studies

**Fig. (1):**
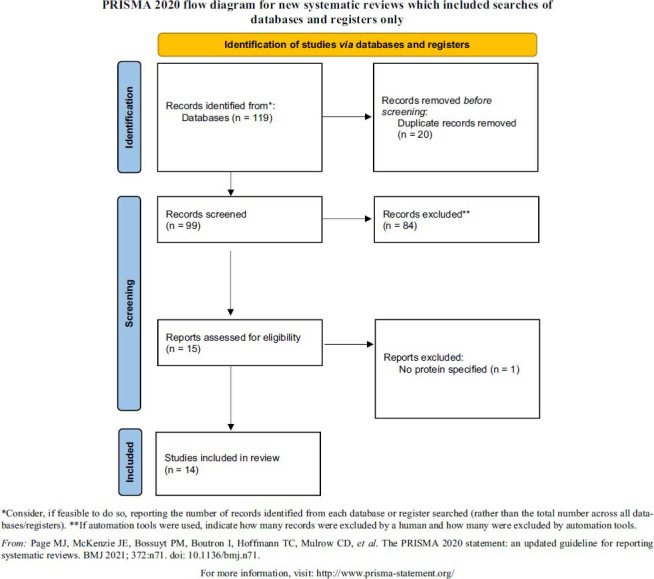
Preferred Reporting Items for Systematic Review (PRISMA) flow chart - search strategy. A total of 14 studies fulfilled the inclusion criteria.

A total of 14 studies fulfilled the inclusion criteria, producing a dataset of numerous candidate protein biomarkers for suicide (Fig. **1**). One paper in which the study did not specify the exact protein detected by proteomic analysis was excluded [18].

### 3.2 Study Characteristics

The following data were extracted from the included studies: the examined element, the used method, the sample size, the main result of the study, and the study source, a summarized in Table **1**.

All of the included studies were prospective, with the exception of the final one, which was a brief report on findings from the proteomic analysis of pre-existing postmortem brain tissue.

### 3.3 Risk of Bias

This systematic review boasts numerous strengths, including its extensive coverage of studies from around the world and the meticulous manual search and examination of reference lists to identify all pertinent studies. Nonetheless, the scarcity of proteomic studies and published papers specifically related to suicide may pose a limitation to the statistical robustness of other potential biomarkers for suicide.

### 3.4 Candidate Protein Biomarkers for Suicide

#### 3.4.1 High Tenascin-C Level

Tenascin-C plays a key role in the development of the hippocampal region [19-21], which has been connected to depression [22, 23]. Furthermore, proteomic studies have reported tenascin-C as a suggested biomarker for major depressive disorder (MDD) [24, 25].

The level of tenascin-C was elevated both in depressed patients and depressed suicide attempters compared to controls, but the highest values were measured in suicide attempters. It is important to note that depressed individuals who made suicide attempts had higher levels of inflammation [26], which may have an impact on the increase of tenascin-C mediated by transforming growth factor-β (TGF-β) [27].

#### 3.4.2 Potassium Voltage-gated Channel Subfamily Q Member 3 (KCNQ3)

Electrical excitation of neurons is facilitated by KCNQ3 [28, 29], and disruption of this protein is associated with neurological conditions, such as benign familial newborn convulsions [30], and psychiatric conditions, such as bipolar disorder and attention deficit hyperactivity disorder [31, 32]. Recent findings suggest a direct connection, indicating a trend of reduced KCNQ3 levels in the dorsolateral prefrontal cortex (DLPFC) of suicide victims [15].

#### 3.4.3 Vimentin-Immunoreactive (VIM-IR) Astrocytes

A broad and steady decline in the density of glial fibrillary acidic protein- immunoreactive (GFAP-IR) and VIM-IR astrocytes was reported in MDD who died of suicide. However, numerous investigations document a decrease in glial densities in samples derived from patients suffering from depression [33, 34]. However, in depressed suicides, vimentin showed a greater reduction than glial densities.

VIM-IR astrocytes showed a widespread reduction in density in cortical areas among depressed suicides. Astrocytes have a crucial nutritionally supportive role for neurons [35]. Vimentin is involved in the regulation of astrocyte structure and in inflammation, which can be compensated by GFAP [36-38].

The widespread decline in these astrocytes in the cerebral cortex, which was reported in depressed suicide, may be connected to the reduced ability to deal with stress [39].

#### 3.4.4 Glutathione S-transferase Theta 1 (GSTT1)

GSTT1 was found to be related to both suicide completion and suicidal risk factors [14]. The polymorphism of the GSTT1 protein, which plays a significant role in coping with oxidative stress [40], has been proposed as a possible biomarker for psychiatric illnesses [41].

Consequently, it could validate the association between psychiatric disorders and apoptosis initiated by neuronal injury resulting from the accumulation of oxidative products [42]. However, being directly related to committing suicide suggests that it could be considered as a biomarker for a higher risk of suicide for psychiatric patients.

#### 3.4.5 Iron Transport Proteins

Iron plays a critical role in numerous pathways within the body. Elevated levels of iron have been implicated in neurodegenerative diseases with psychiatric manifestations [43, 44]. Conversely, iron deficiency has been linked to psychiatric diseases [45]. Several studies have highlighted the involvement of iron transport proteins specifically in the pathophysiology of psychiatric conditions such as MDD [46] and schizophrenia [47].

However, alteration of these iron transport proteins levels in suicide with no known history of psychiatric disorder makes them considered potential biomarkers for making the suicide decision and committing it.

#### 3.4.6 Crystallin Chain B (CRYAB), Manganese Superoxide Dismutase (SOD2), and Glial Fibrillary Acidic Protein

Among the heat shock proteins with low molecular weight is CRYAB. It is a protective protein that keeps GFAP from becoming inactive in astrocytes [48]. CRYAB builds up in astrocytes when a neurodegenerative disease occurs [49], like in the cases of Alzheimer's [50, 51] and multiple sclerosis [52]. The elevation of CRYAB was also found in suicide, suggesting its possible role as a biomarker [5].

A decrease in SOD2 may indicate that the cortical tissue is unable to cope with oxidative stress, as this protective protein guards against tissue damage caused by it. Its rise is explained by the body's attempt to compensate for the stress. However, different levels of SOD2 were detected in neurological diseases and psychiatric disorders in different studies [53-56], making it hard to elucidate its importance. However, SOD2 was up-regulated in the cerebral tissue of suicide victims when compared to a control group.

GFAP, or astroglia marker, has been extensively investigated for its level and various isoforms, and it is thought to be a biomarker for neurological and psychiatric disorders [57, 58]. The phosphorylated form, which is the most significant in mental illness, was discovered only in the brain samples of suicide victims when compared to a control group.

#### 3.4.7 Enolase 1 (ENO1), Enolase 2 (ENO2), Glyceraldehyde-3-phosphate (G3P), Aldolase C (ALDOC), Peroxiredoxin 2 (PRDX2)

These proteins are involved in the glycolytic pathway and are responsible for glucose utilization [59, 60]. The downregulation of these proteins suggests a reduced use of glucose in the central nervous system (CNS) of individuals who commit suicide. Additionally, the significant upregulation of ALDOC in the glycolysis/gluconeogenesis pathway can be related to an increase in the demand for glucose as a compensation mechanism [11].

PRDX2 and ENO2 are part of the oxidative stress response, with ENO2 also serving as a marker of nervous tissue damage. The downregulation of both proteins in suicidal cases may indicate a decrease in the oxidative stress response [61].

#### 3.4.8 Protein 14-3-3 eta (YWHAH) and 14-3-3 theta (YWHAQ)

YWHAH and YWHAQ 14-3-3-mediated signaling pathways were significantly upregulated in the CSF of suicidal individuals [11].

These findings contrast with a study that identified the upregulation of YWHAH, but not YWHAQ, in the prefrontal cortex and amygdala in suicidal attempters. Interestingly, the protein 14-3-3 epsilon (YWHAE) was elevated in both suicidal cases and controls despite the gene responsible for coding this protein being previously associated with suicidality [62]. The upregulation of these proteins is interpreted as a neuroprotective process against specific neurodegenerative diseases, and neurodegeneration is found to have a role in suicide [63].

The 14-3-3 proteins are a family of adaptor proteins that regulate enzymatic activity, maintain protein structure, and enhance protein stability upon binding in a large complex, participating in all cellular biological activities [64-66].

#### 3.4.9 Specific Cytoskeleton Proteins

Alpha-internexin (INA), neurofilament light polypeptide 68 KDa (NEFL), neurofilament medium polypeptide (NEFM), tubulin alpha-1B chain, creatine kinase B-type, and heat shock cognate 71 KDa protein showed upregulation in both the prefrontal cortex and amygdala in suicidal cases. Cathepsin D, actin cytoplasmic 1, and GFAP showed downregulation in the cortex and upregulation in the amygdala.

The protein interaction network of these proteins contained a direct interaction sub-network of cytoskeleton proteins (INA, NEFL, NEFM, and GFAP) that is connected to the network of glutamate and serotonin receptors involved in psychiatric illnesses through GRIN1 (Glutamate Ionotropic Receptor NMDA Type Subunit 1) [67].

#### 3.4.10 C-reactive Protein (CRP), Serum Amyloid A Protein 1 (SAA1), Extrinsic Coagulation Pathway Proteins

The relationship between suicide, stress, and depression leads to increased levels of inflammatory cytokines [68-71]. CRP and SAA1 are acute-phase reactants that increase in response to inflammatory cytokines. These reactants also participate in the coagulation process by inducing monocytes to express cell-bound tissue factor (TF) [72].

Patients with MDD, both attempters and non-attempters, showed a proinflammatory-hypothrombotic state with higher levels of CRP and SAA1, lower levels of factor 1+2, and lower prothrombinase activity compared to healthy controls. Additionally, MDD patients who attempted suicide showed a more proinflammatory-prothrombotic state than MDD patients who did not attempt suicide, with significantly higher CRP and SAA1 levels, higher relative prothrombinase activity, and increased levels of TF, FVII, FX, FV, and F1+2.

#### 3.4.11 The Subunit of Vacuolar-type Proton Pump ATPase (VPP1)

VPP1 is part of a multi-subunit complex called the vacuolar proton pump ATPase, which is responsible for moving protons through the cellular membrane. VPP1 is an important component of synaptic vesicles, maintaining the pH gradient and membrane potential necessary for storing neurotransmitters and releasing them into the synaptic cleft [73-75].

It was found that the protein level of VPP1 in the cerebellum was downregulated in cases of suicide but not in elderly schizophrenia cases without suicide. This suggests a link between VPP1 and suicidal behavior, which is often associated with dysregulation of synaptic activity, particularly in the serotonergic pathway [76, 77].

Another study found VPP1 downregulation in the anterior cingulate cortex in younger schizophrenic patients, some of whom were suicidal [78]. Additionally, a separate study revealed alterations in VPP1 in the frontal cortex of patients with major depressive disorder who committed suicide [77].

#### 3.4.12 Alteration of Plasma Apolipoprotein A-IV (Apo A-IV)

Downregulation of Apo A-IV was noted in the plasma of suicidal attempters [79]. This alteration of Apo A-IV was found to be the cause of the decreased activity of cholesterol acyltransferase (LCAT) [80], an enzyme that converts cholesterol to esterified cholesterol [81]. As a result, the level of esterified cholesterol decreased, which affected the ratio between esterified cholesterol and free cholesterol. This altered ratio could increase the viscosity of the cell membrane and affect the neurotransmission process, mainly serotonin [82].

Another explanation for the role of altered Apo A-IV is the direct effect of cholesterol on membrane integrity caused by decreased activity of LCAT. Also, the effect of disturbed cholesterol on the permeability of the blood-brain barrier was also reported in suicidal attempters [83].

In contrast to a study on the association between plasma ApoE and suicide attempts, which found no alterations in plasma levels of ApoE between suicide attempters and healthy controls [84], another study revealed an upregulation of ApoE in individuals with repeated suicidal attempts compared to those who had never attempted suicide before. Higher levels of ApoE were also found in those with a high number of previous attempts. After accounting for age, there was a substantial negative correlation between age at onset and ApoE. In male suicide attempters, ApoE showed a strong positive association with early exposure to interpersonal violence. These results suggest that the temporal severity of suicidal behavior and ApoE may be associated with stress and trauma [85].

The relationship between ApoE and the severity of suicidal behavior was also reported. CSF ApoE was upregulated in individuals with repeated suicidal attempts (at least one previous attempt) compared to those who had never attempted suicide before. In addition, CSF ApoE was downregulated in cases of violent suicidal attempts [86].

#### 3.4.13 Dysregulation of Endoplasmic Reticulum (ER) Stress Proteins

A significant increase in the levels of glucose-regulated protein (GRP78, GRP94) and calreticulin was noted in the temporal cortex of suicidal cases of MDD compared to MDD patients who died due to other causes [87].

Suicidal cases in patients with bipolar or schizophrenia showed no alteration in these proteins. Therefore, suicide itself may not be related to this alteration. GRP78 is a part of the ER stress protein family, which includes both GRP94 and calreticulin. These proteins are calcium-binding proteins and have a role in the regulation of protein folding [88]. Being considered neuroprotective proteins, their increase in neurodegenerative conditions is most likely a compensatory mechanism [89].

## 4 DISCUSSION

Numerous studies and reviews discuss the different biomarkers in psychiatric illnesses in terms of diagnosis, prognostic follow-up, and management [90, 91]. Moreover, several studies consider the genomic aspect of suicide [92, 93]. While it is established that the majority of psychiatric diseases raise the risk of suicide [94, 95], the majority of patients with psychiatric disorders never attempt suicide [96, 97]. Upon conducting a search on the potential changes in proteins linked to suicide, a dearth of papers was found, the majority of which focused on suicide in specific mental conditions, that could have affected the specificity of altered proteins. Therefore, further investigation in this area is needed. The sample should solely consist of people who have either completed or attempted suicide and without any prior history of psychiatric disorders. If such disorders are present, then common biomarkers both for suicide-associated psychiatric disorders and non-suicidal psychiatric disorders should be excluded.

Different proteins related to different biological processes and pathways were reported to be altered in suicidal cases [14]. The effect of these alterations in suicide is mostly related to the central nervous system (CNS).

Stress, a well-known risk factor for suicide, can dysregulate glucose utilization and lead to alterations in glycolytic proteins (ENO1, ENO2, G3P, and ALDOC) [11].

Additionally, stress and depression can increase the release of inflammatory cytokines, causing alterations in proteins related to inflammatory and coagulation responses (CRP, SAA1, TF, FVII, FX, FV, TFPI, APC, PCI, and F1+2) [68].

Neurodegeneration, another risk factor for suicide, is associated with the upregulation of certain proteins (YWHAH, YWHAQ, PRDX2, CRYAB, GRP78, GRP94, and calreticulin) that have a neuroprotective effect [5, 11]. Reduced levels of certain proteins (Astrocytic immunoreactive type of GFAP and VIM) may be due to the loss of astrocytes, which has been reported in depressed individuals [33].

The dysregulation of neurotransmitter signaling pathways, particularly serotonergic, glutamatergic, and GABAergic pathways, may be influenced by altered proteins (cytoskeleton proteins, VPP1, and Apo A-IV), which can play a role in suicide susceptibility [16, 73, 79]. The role of neurotransmitters, especially serotonergic, and their signaling pathways in the liability and susceptibility to suicide has been extensively discussed in the literature [98, 99].

The accumulation of oxidative stress due to decreased levels of numerous antioxidant proteins (PRDX2, ENO2, GSTT1, SOD2) in the CNS can result in neuronal injury, providing a possible explanation for the role of these proteins in suicide [5, 11, 14, 87]. Furthermore, hippocampal dysfunction may be related to the increased level of Tenascin C [5]. Although the connection between the hippocampus and suicidality is unclear, it has been discussed in the literature [100, 101].

Neuronal electrical abnormality can be a physiological factor for suicide, potentially explaining the downregulated level of the excitatory protein KCNQ3 in suicidal cases [15]. Additionally, increased levels of iron transport proteins in the cortex of suicidal cases, which are related to the increased level of iron, can contribute to multiple neurodegenerative diseases [43].

Assessing the risk of suicide is crucial for prevention efforts. Improving the understanding of proteins associated with suicide, particularly those measurable in the blood of living individuals, is essential. In plasma samples, the levels of several biomarkers can serve as useful indicators for assessing suicide risk. These include Tenascin C, C-reactive protein, serum amyloid A protein 1, extrinsic coagulation pathway proteins, apolipoprotein A-IV, and apolipoprotein E. Monitoring these biomarkers may help in identifying individuals at higher risk of suicide. Combining this protein data with other omics information can enhance the predictive value of suicide risk assessment. This holistic approach can lead to more effective prevention strategies.

Moreover, it can serve as a valuable tool for forecasting suicide risk in cases where determining the manner of death is critical.

While this study provides valuable insights into candidate proteins as biomarkers for predicting suicides, it highlights the need for further research to enhance understanding and diagnostic approaches. Future studies should involve larger sample sizes to improve the statistical power and generalizability of the findings. Additionally, incorporating a broader range of study specimens, including diverse populations and different biological samples, can provide a more comprehensive view of this. By expanding research efforts in these directions, more accurate diagnostic tools and effective interventions can be developed, ultimately advancing the field and improving outcomes.

## 5 LIMITATIONS

- The comprehensiveness of the review is constrained by the exclusion of non-English language studies and the presence of missing or incomplete data in the primary studies.
- There are few studies specifically investigating proteomics in individuals who have completed or attempted suicide.
- The primary focus on brain tissue as a protein source limits applicability, as this method cannot be used for living individuals.
- The review is limited by the heterogeneity among the studies that met the inclusion criteria. This variability may affect the consistency and comparability of the findings.

## CONCLUSION

In conclusion, suicide is a global concern impacting both psychiatric patients and the general population. While much research has been dedicated to diagnosing and treating psychiatric disorders, there has been relatively little focus on predicting and preventing suicide. This review consolidates proteomic studies conducted on suicidal cases, aiming to address this gap and potentially identify biomarkers for suicide. Such findings can pave the way for the development of more effective preventive approaches.

## Figures and Tables

**Table 1:** Proteomic analyses of suicide cases.

**S. No.**	**References**	**Examined Element**	**Used Method**	**Number of Samples**	**Main Results**
1	Translational Psychiatry, 12(1), 142.	The prefrontal cortex - Brodmann’s area 9 and 10	Liquid chromatography-tandem mass spectrometry	Thirty six cases: 23 suicides and 13 controls.	The study revealed significantly elevated levels of endocannabinoid and apoptotic pathway markers (CAPNS1, CSNK2B, and PTP4A2) ^1^ in samples from individuals who died by suicide. Additionally, GSTT1 ^2^, which displayed a significant difference, was associated with both completed suicide and risk factors for suicide.
2	Frontiers in Psychiatry, 12, 640963.	The mediodorsal thalamus, dorsal caudate nucleus, and dorsomedial prefrontal cortex	Immunohistochemistry	Twenty adults; half of them committed depressed suicide.	The study indicated a widespread decrease in VIM-IR ^3^ astrocyte density across cortical areas in depressed individuals who died by suicide.
3	Genes, 11(3), 256.	The dorsolateral prefrontal cortex	Mass spectrometry	Ten cases of mood disorder; half of them committed suicide.	Among the 5162 proteins identified, only 33 exhibited significantly altered expression in suicide cases. Notably, KCNQ3 ^4^, the most markedly affected protein, displayed reduced expression in samples from suicide patients.
4	PloS one, 15(7), e0230400.	Cerebellar grey matter	Mass spectrometry	The study examined four schizophrenic patients who died by suicide and four cases as controls. Subsequently, the identified altered proteins were validated by comparing them to non-schizophrenic suicide cases and schizophrenic patients who died of natural causes.	The study uncovered a downregulation of cerebellar vacuolar-type proton pump ATPase subunit levels in cases of suicide. Additionally, EF-hand calcium-binding proteins in the cerebellum, such as parvalbumin and calmodulin, exhibited alterations in schizophrenia-related suicide cases but not in non-schizophrenic suicide cases.
5	The World Journal of Biological Psychiatry, 21(2), 119-126.	Brodmann’s area 6 and 10	Tissue processing and Western blot analyses	The study comprised 38 cases, including 13 patients with major depressive disorder, 12 with bipolar disorder, and 13 controls.	The study examined various iron transport proteins, such as ferritin, Cp, TAU, PrPC, APP ^5^, and the transferrin receptor. It discovered elevated levels of transferrin, TAU, and amyloid precursor protein in Brodmann’s area 10 among suicide cases. Conversely, copper-containing ceruloplasmin levels were lower in Brodmann’s area 6 within the same group.
6	Indian Journal of Medical Research, 150(4), 365-375.	Plasma	Two-dimensional gel electrophoresis coupled with matrix-assisted laser desorption-ionization mass spectrometry	The study comprised two phases: the discovery phase and the validation phase. In the discovery phase, ten patients with deliberate self-harm were compared to ten controls. Subsequently, in the validation phase, eighteen patients with deliberate self-harm were compared to thirteen matched controls.	The study findings indicated a downregulation of apolipoprotein A-IV in individuals who had attempted suicide.
7	Journal of Affective Disorders, 225, 246-249.	Cerebrospinal fluid (CSF)	Immunonephelometry	There were 42 living individuals, all medication-free, who had recently attempted suicide.	CSF apolipoprotein E (ApoE) was upregulated in individuals with a history of repeated suicidal attempts (at least one previous attempt) compared to those who had never attempted suicide before. Additionally, CSF ApoE was downregulated in cases of violent suicidal attempts. The presence of ApoE in CSF may be associated with the irreversibility of suicide attempts.
8	J Proteomics Bioinform, 11, 117-119.	Cerebrospinal fluid	Mass spectrometry	The study included six individuals: two middle-aged males who died by suicidal hanging without medical history or medication, two middle-aged males who succumbed to heart disease without medical history or medication, and two females with hydrocephalus and medication history.	Several glycolytic proteins, namely enolase 1, enolase 2, glyceraldehyde-3-phosphate, and triose phosphate isomerase, were notably down-regulated in the CSF of individuals who died by suicide. Additionally, there was a significant upregulation of protein 14-3-3 eta and 14-3-3 theta, indicating an activated 14-3-3 mediated signaling pathway in suicidal cases compared to both cardiac and hydrocephalus cases.
9	Psychiatry Research 268 (2018): 60-64.	Fasting blood samples	Immunosorbent assay	Two hundred thirty eight subjects - 129 depressive patients and 109 controls.	Patients' suicide attempts and depression severity correlated positively with high Tenascin C levels.
10	Journal of Affective Disorders, 190, 137-142.	Plasma	Immunonephelometry	Included were 100 suicidal attempters, comprising 67 females and 33 males.	The study unveiled an upregulation of Apolipoprotein E (ApoE) in individuals with a history of repeated suicidal attempts compared to those who had never attempted suicide previously. Additionally, ApoE was suggested to be associated with stress, trauma, and the temporal severity of suicidal behavior.
11	Scientific Reports, 6(1), 32882.	Blood samples	Western blot analyses	Three groups were studied, each comprising 12 individuals. These groups encompassed patients with major depressive disorder (MDD) who attempted suicide (MDD-SA), patients with MDD who did not attempt suicide, and controls. Additionally, ELISA ^6^ validation was performed on other related proteins for the same groups, with each group comprising 49 individuals.	Ten proteins were identified as altered in the plasma of MDD-SA patients. These included two inflammatory proteins, C-reactive protein and serum amyloid A1, as well as eight coagulation proteins: tissue factor, coagulation factor VII, coagulation factor X, coagulation factor V, tissue factor pathway inhibitor, activated protein C, protein C inhibitor, and prothrombin fragment (fragment 1+2).
12	PloS One, 7(12), e50532.	Prefrontal cortex and amygdala	Western blot analyses	Twelve cases: half of them committed hanging and half of them as controls.	In individuals who died by suicide, numerous cytoskeleton proteins, such as Alpha-internexin, Neurofilament (light polypeptide 68 kDa), Neurofilament (medium polypeptide), Tubulin alpha-1B chain, Creatine kinase B-type, and Heat shock cognate 71 kDa protein, exhibited upregulation in both the prefrontal cortex and amygdala. Conversely, Cathepsin D, Actin cytoplasmic 1, and Glial fibrillary acidic protein demonstrated downregulation in the cortex but upregulation in the amygdala.
13	Journal of Psychiatric Research, 41(6), 493-501.	Prefrontal cortex (Brodmann’s area 10) and cerebellar tissues	Two-dimensional gel electrophoresis	Out of 26 subjects, 17 died of suicide.	After excluding protein degradation, five proteins exhibited significant differences, three of which were exclusive to suicide victims and absent in the control group: manganese superoxide dismutase, glial fibrillary acidic protein, and crystallin chain B, with the latter showing the most pronounced differences in spot intensities.
14	Neuropsychopharmacology, 22(3), 327-332.	Temporal cortex	Immunoblotting	There were four groups, each comprising fifteen individuals matched for age and sex. These groups included individuals with bipolar disorder (BD), individuals with major depressive disorder (MDD), individuals with schizophrenia (SCZ), and controls.	In the temporal cortex of MDD cases who died by suicide, there was a notable increase in the levels of the 78-kilodalton glucose-regulated protein, glucose-regulated protein 94, and calreticulin compared to MDD patients who died due to other causes. However, patients with BD or SCZ who died by suicide showed no alteration in these proteins.

**Abbreviations:** ^1^CAPNS1: Calpain small subunit 1. CSNK2B: Casein kinase 2 beta. PTP4A2: protein tyrosine phosphatase 4A2, ^2^GSTT1: Glutathione S-transferase theta 1, ^3^VIM-IR: Vimentin-Immunoreactive Astrocytes, ^4^KCNQ3: Potassium Voltage-Gated Channel Subfamily Q Member 3, ^5^CP: Ceruloplasmin. PrPC: Prion protein. APP: Amyloid precursor protein, ^6^ELISA: The enzyme-linked immunosorbent assay.

## LIST OF ABBREVIATIONS

CNS: Central Nervous System
CRP: C-reactive Protein
CRYAB: Crystallin Chain B
DLPFC: Dorsolateral Prefrontal Cortex
ER: Endoplasmic Reticulum
GSTT1: Glutathione S-transferase Theta 1
MDD: Major Depressive Disorder
SOD2: Superoxide Dismutase
TF: Tissue Factor

## STANDARDS OF REPORTING

PRISMA guidelines and methodology were followed.

## SUPPLEMENTARY MATERIAL

PRISMA checklist is available as supplementary material on the publisher’s website along with the published article.
